# Health-related social media engagement and cervical cancer prevention: associations with HPV knowledge, vaccine awareness, and pap smear utilization among US women

**DOI:** 10.3389/fpubh.2026.1782691

**Published:** 2026-07-07

**Authors:** Osamah M. Al-Omair, Noof S. Alabdullatif

**Affiliations:** 1 Department of Management Information Systems, College of Business Administration, King Faisal University, Al-Ahsa, Saudi Arabia; 2 Johns Hopkins Aramco Healthcare, Dhahran, Saudi Arabia

**Keywords:** cervical cancer screening, health communication, health disparities, health-related social media, HPV vaccine awareness, human papillomavirus, preventive health behavior, women’s health

## Abstract

**Background:**

Persistent infection with human papillomavirus (HPV) is the primary cause of cervical cancer, a largely preventable disease through HPV vaccination and Pap smear screening. However, gaps remain in HPV knowledge, vaccine awareness, and screening utilization among women in the US. Social media has become a common source of health information and may influence preventive health behaviors, yet evidence linking health-related social media engagement to HPV-related outcomes is limited.

**Methods:**

Data were drawn from the Health Information National Trends Survey (HINTS) 6, a nationally representative survey of US adults. The analytic sample included 2,247 women aged 21–65 years. Ordered logistic regression was used to examine HPV knowledge, while logistic regression models assessed HPV vaccine awareness and Pap smear utilization, adjusting for sociodemographic, socioeconomic, and health-related covariates.

**Results:**

Women who engaged with health-related content on social media had higher odds of greater HPV knowledge (OR = 1.75, 95% CI: 1.43–2.13) and HPV vaccine awareness (OR = 1.60, 95% CI: 1.25–2.05). Health-related social media engagement was also associated with increased Pap smear utilization (OR = 1.26, 95% CI: 1.00–1.59). Increasing levels of health-related social media engagement showed significant linear dose–response associations with HPV knowledge, HPV vaccine awareness, and Pap smear utilization among US women.

**Conclusion:**

These findings highlight the potential of social media–based interventions to support cervical cancer prevention efforts, while underscoring the need for culturally tailored strategies to reduce persistent disparities across racial and ethnic groups.

## 1 Introduction

Cervical cancer remains a major yet largely preventable public health problem worldwide and in the US. It ranks as the fourth most common cancer among women worldwide, with approximately 660,000 new cases and 350,000 deaths reported in 2022 (1). In the US, widespread use of screening and vaccination has reduced the burden, but an estimated 13,360 new cases and more than 4,320 deaths are still expected in 2025 (2). Early detection is a critical component of cervical cancer prevention strategies. Utilization of screening tools such as the Papanicolaou (Pap) smear and human papillomavirus (HPV) testing facilitates the timely identification and management of premalignant cervical lesions, thereby mitigating progression to invasive carcinoma (2). The US Preventive Services Task Force recommends routine cervical cancer screening for women aged 21 to 65 years, with options including Pap testing every 3 years or Pap and HPV co-testing every 5 years for women aged 30 to 65 years (3).

Despite strong evidence and clear guidelines, screening utilization remains suboptimal, especially among racial and ethnic minorities and people with lower socioeconomic status, which contributes to persistent disparities in cervical cancer incidence and mortality (4, 5). Limited awareness of screening recommendations, low health literacy, and barriers related to access and trust all play a role, leaving many women unaware of when and why they should be screened (6). Expanding education, improving access, and tailoring outreach to underserved communities are essential to increase screening participation and reduce preventable cervical cancer morbidity and mortality across all populations.

Racial and ethnic disparities in cervical cancer remain a significant public health concern in the US Black women face a 30% higher risk of developing cervical cancer and a 60% higher mortality rate compared to non-Hispanic White women, while Hispanic women experience about a 50% higher incidence and approximately 20% higher mortality than White women (5, 7, 8). These disparities persist despite overall declines in cervical cancer due to screening and HPV vaccination and are largely driven by inequities in access to prevention, early detection, and timely treatment services (8, 9). Pap smear utilization also varies significantly by race and ethnicity, with Black and Hispanic women less likely to receive recommended co-testing with HPV and more likely to experience delays or inadequate follow-up after abnormal results (5, 10). Furthermore, social media usage as a health promotion tool varies across racial and ethnic groups, with differences in access, digital literacy, and trust potentially influencing the reach and effectiveness of social media-based screening campaigns (11). Addressing these disparities requires targeted strategies that acknowledge and overcome social, economic, and structural barriers to cervical cancer screening and care in underserved populations (5, 9).

Social media has become a central channel for health promotion, allowing organizations and individuals to rapidly share information, counter misinformation, and tailor messages to specific populations through platforms such as Facebook, Instagram, X (Twitter), TikTok, and WhatsApp (12). Evidence from multiple cancer and prevention campaigns shows that well-planned social media efforts can increase health knowledge, shift attitudes, and sometimes improve participation in screening and vaccination (13). However, application to cervical cancer screening specifically remains underexamined.

Current evidence on social media and cervical cancer screening is still limited, leaving several important gaps in knowledge (14, 15). Existing studies mainly examine general associations between online health information, HPV awareness, or cancer screening behaviors, with relatively few focusing specifically on how social media affects cervical cancer screening awareness and actual utilization of Pap or HPV testing (11, 14). Moreover, most available work does not assess whether the impact of social media varies by key demographic and socioeconomic characteristics such as age and race/ethnicity, so potential effect modification across population subgroups remains largely untested (11, 15). This lack of analyses limits understanding of which groups benefit most, who may be left behind, and whether social media–based interventions reduce existing disparities in cervical cancer screening (15).

These gaps are particularly concerning given that, despite substantial declines in cervical cancer incidence due to screening and HPV vaccination, persistently low screening utilization especially among disadvantaged populations continues to drive disparities. Health-related social media engagement may serve as a valuable channel for disseminating cervical cancer–related information and supporting preventive health behaviors; however, evidence remains limited regarding its association with HPV knowledge, HPV vaccine awareness, and Pap smear utilization. Using nationally representative US data, this study addresses these gaps by examining the associations between health-related social media engagement and HPV knowledge, HPV vaccine awareness, and Pap smear utilization among women aged 21–65 years. We further assess whether these associations follow a dose–response pattern. In addition, we examine racial and ethnic disparities in these outcomes by assessing whether race/ethnicity modifies the associations between social media engagement and HPV knowledge, HPV vaccine awareness, and Pap smear utilization.

## 2 Materials and methods

### 2.1 Study population and design

This study was a secondary analysis of data from the Health Information National Trends Survey (HINTS) 6, a nationally representative survey of non-institutionalized adults in the United States conducted by the National Cancer Institute in March 2022. HINTS 6 uses a probability-based, address-stratified sampling design and collects data via mixed-mode surveys (web and paper). Households were randomly assigned to either a concurrent mode (both modes offered simultaneously) or a sequential mode (web offered first, paper offered later). One adult per household was selected using the Next Birthday Method, and survey weights were provided to generate nationally representative estimates. Surveys were available in English and Spanish, and all participants received a small monetary incentive to encourage participation. For more details on the HINTS 6 methodology, sampling, and weighting procedures, refer to the official HINTS documentation (16).

The analytic sample was restricted to 2,247 women aged 21–65 years, reflecting age groups relevant for HPV vaccination, HPV knowledge, and cervical cancer screening. Participants with missing data on the primary exposure (health-related social media engagement) or outcomes (HPV knowledge, HPV vaccine awareness, and Pap smear utilization) were excluded. The study was conducted in accordance with the Declaration of Helsinki. It used publicly available, de-identified data and was therefore exempt from institutional review board (IRB) approval and informed consent procedures. No identifiable information was used in the analysis.

### 2.2 Measures

#### 2.2.1 Outcomes

This study examined three primary outcomes related to cervical cancer prevention and HPV: HPV knowledge, HPV vaccine awareness, and Pap smear utilization. These outcomes were selected to capture multiple aspects of women’s awareness, preventive behaviors, and engagement with screening guidelines.

HPV knowledge was assessed using two binary HINTS items: “Have you ever heard of HPV? HPV stands for Human Papillomavirus. It is not HIV, HSV, or herpes” and “Do you think HPV can cause cervical cancer?,” the latter asked only of respondents who had heard of HPV. A scale ranging from 0 to 2 was created by summing the responses to these two items, with higher scores indicating greater knowledge. Respondents who answered both items correctly received a score of 2, those who answered one correctly received a score of 1, and those who answered neither correctly received a score of 0. For analysis, this score was used to represent HPV knowledge, capturing both basic awareness of HPV and understanding of its role in causing cervical cancer.

HPV vaccine awareness was measured using a binary item: “A vaccine to prevent HPV infection is available and is called the cervical cancer vaccine or HPV shot. Before today, have you ever heard of the cervical cancer vaccine or HPV shot?”

Pap smear utilization was assessed using the HINTS item “When was your last Pap screen?” Response options included: a year ago or less, more than 1 up to 2 years ago, more than 2 up to 3 years ago, more than 3 up to 5 years ago, more than 5 years ago, and I have never had a Pap test. For this study, utilization was dichotomized to reflect guideline-recommended screening, with respondents reporting a Pap test within the past 3 years or less coded as (yes) and all others coded as (no) based on current cervical cancer screening recommendations for women aged 21–65 years.

#### 2.2.2 Exposures

The primary exposure was health-related social media engagement, assessed using four HINTS items asking participants about their social media activities in the past 12 months:

- “How often did you share personal health information on social media?”
- “How often did you share general health-related information on social media (for example, a news article)?”
- “How often did you interact with people who have similar health or medical issues on social media or online forums?”
- “How often did you watch a health-related video on a social media site (for example, YouTube)?”

Response options for each item included: almost every day, at least once a week, a few times a month, less than once a month, or never. A composite binary variable was created to indicate engagement with health-related social media. Participants were coded as 1 (engaged) if they reported any frequency of participation other than “Never” on at least one of the four items, and 0 (not engaged) if they reported “Never” on all items. This composite captures overall interaction with health information on social media and online platforms. This binary operationalization was used to facilitate a clear primary comparison between individuals with any exposure to health-related social media and those with no exposure, consistent with prior epidemiological approaches. To further account for heterogeneity in engagement behaviors, a supplementary variable capturing the extent of engagement was also constructed and analyzed.

A second exposure variable was constructed to capture the extent of health-related social media engagement. This was operationalized as a count variable (range 0–4), generated by summing the number of engagement activities reported as occurring at any frequency other than “never” across the four items. Higher scores indicated a greater breadth of engagement with health-related social media behaviors and were used to examine dose–response relationships.

#### 2.2.3 Confounding variables

Several variables were included as covariates because they may influence HPV outcomes, Pap smear utilization, and engagement with health-related social media, and have been commonly used in previous research. Age was categorized into 21–29, 30–39, 40–49, and 50–65 years. Race/ethnicity included non-Hispanic White, Hispanic, non-Hispanic Black, non-Hispanic Asian, and non-Hispanic Other. Marital status was classified as married or unmarried. Health insurance coverage included private, public, military, other, and uninsured.

Employment status was categorized as employed, unemployed, homemaker/student/retired/disabled, multiple occupation statuses, or other. Household income was grouped into $0–49,999, $50,000–99,999, $100,000–199,999, and $200,000 or more. Educational attainment included less than high school, high school/some college, or college and above. Self-reported health status was categorized as poor/fair, good, or very good/excellent. Region of residence included Northeast, Midwest, South, or West.

### 2.3 Statistical analysis

Descriptive statistics summarized participant characteristics, with frequencies (N) and percentages (%) for categorical variables, and means with standard deviations (SD) for continuous variables. Separate multivariable regression models were constructed for each outcome to identify independent associations with health-related social media engagement while adjusting for age, race/ethnicity, marital status, region, health insurance coverage, employment status, education, household income, and self-reported health status.

HPV knowledge was analyzed as an ordinal outcome using ordinal logistic regression based on the HPV knowledge scale (range 0–2), as shown in Equation 1:


$$\text{logit}[P(Y_{i}\leq m)]=\alpha_{m}-\sum_{k=1}^{p}\;\beta_{k}X_{\mathrm{ik}}m=0,1$$

For binary outcomes (HPV vaccine awareness and Pap smear utilization), logistic regression models were used as shown in Equation 2:

(2) $$\text{logit}[P(Y_{i}=1)]={𝛽}_{0}\sum_{k=1}^{p}\;\beta_{k}X_{\mathrm{ik}}$$ (1)

Adjusted odds ratios (ORs) with 95% confidence intervals (CIs) were reported for all models. To assess potential effect modification by race/ethnicity in the association between health-related social media engagement and each outcome (HPV knowledge, HPV vaccine awareness, and Pap smear utilization), race/ethnicity was included as an interaction term in the regression models, with non-Hispanic White specified as the reference group. Statistical significance was defined as a *p*-value < 0.05. Missing data were minimal and handled using complete case analysis: 0.76% for HPV knowledge, 1.33% for HPV vaccine awareness, and 2.77% for Pap smear utilization.

All analyses accounted for the complex survey design of the Health Information National Trends Survey (HINTS). Sampling weights, strata, and primary sampling units (PSUs) were applied in all descriptive and regression analyses to ensure nationally representative estimates. Variance estimation was adjusted for the complex sampling design in all models.

All analyses were performed using STATA version 16 (StataCorp, College Station, TX). Strengthening the Reporting of Observational Studies in Epidemiology Statement checklist (STROBE) was followed (17).

## 3 Results

### 3.1 Characteristics of study participants

Table 1 presents the sociodemographic and health characteristics of the study sample, which included 2,247 women aged 21 to 65 years. The average age of participants was 47.03 years. The majority identified as non-Hispanic White (50.96%), followed by Hispanic (21.99%) and non-Hispanic Black (18.27%). Most participants were unmarried (56.28%), insured (88.93%), and employed (61.62%). About half of the sample (49.26%) had completed college degree or higher. Household income varied, with 42.19% earning less than $50,000 annually. In terms of health status, 37.47% reported being in “good” health, with smaller proportions reporting “very good” (33.97%) or “excellent” (10.25%) health. The largest proportion of participants resided in the South (43.44%), with fewer from the West (23.50%), Midwest (17.62%), and Northeast (15.44%). Overall, 73.52% of women reported engaging in at least one health-related social media activity. While the majority did not share personal (81%) or health-related (64%) information, and 71% did not interact with peers facing similar health issues, a higher proportion (66%) reported watching health-related videos online (Supplementary Table S1).

**Table 1:** Sociodemographic, health, and social media engagement characteristics of women aged 21–65 in the study sample (*n* = 2,247).

Variable	Frequency (N), percentage (%)	Mean (SD)
Age (Continuous)		47.03 (12.52)
Age (Categorical)		
21–29	250 (11.13)	
30–39	467 (20.78)	
40–49	431 (19.18)	
50–65	1,099 (48.91)	
Race/ethnicity		
Non-Hispanic White	1,110 (50.96)	
Hispanic	398 (21.99)	
Non-Hispanic Black	479 (18.27)	
Non-Hispanic Asian	107 (4.91)	
Non-Hispanic Other	84 (3.86)	
Marital status		
Unmarried	1,260 (56.28)	
Married	979 (43.72)	
Insurance coverage		
Uninsured	248 (11.07)	
Insured	1992 (88.93)	
Employment status		
Unemployed	139 (6.19)	
Employed	1,384 (61.62)	
Homemaker, Student, Retired, Disabled	512 (22.80)	
Multiple Occupation statuses	190 (8.46)	
Other Occupation	21 (0.93)	
Household income quantiles		
$0–$49,999	948 (42.19)	
$50,000–$ 99,999	668 (29.73)	
$100000–$199,999	441 (19.63)	
$200,000+	190 (8.46)	
Education		
Less than high school	135 (6.02)	
High school/ some college	1,002 (44.71)	
College and above	1,104 (49.26)	
Health status		
Poor/Fair	409 (18.31)	
Good	837 (37.47)	
Very good/Excellent	988 (44.23)	
Region		
Northeast	347 (15.44)	
Midwest	396 (17.62)	
South	976 (43.44)	
West	528 (23.50)	
Health-related social media engagement		
No	595 (26.48)	
Yes	1,652 (73.52)	

### 3.2 Associations between HPV knowledge, vaccine awareness, and health-related social media engagement

An ordered logistic regression model was used to examine HPV knowledge, and a logistic regression model was used for HPV vaccine awareness (Table 2). In the adjusted models, women who engaged with health-related content on social media had significantly higher odds of HPV knowledge (OR = 1.75, 95% CI: 1.43–2.13, *p* < 0.001) and HPV vaccine awareness (OR = 1.60, 95% CI: 1.25–2.05, *p* < 0.001) compared to those who did not. The unadjusted regression models showed similar significant associations (*p* < 0.001; Supplementary Table S2). Higher educational attainment was strongly associated with both outcomes. Compared with women who had less than a high school education, those with a college education or higher had 3.36 times higher odds of HPV knowledge (OR = 3.36, 95% CI: 2.25–5.01, p < 0.001) and 2.46 times higher odds of HPV vaccine awareness (OR = 2.46, 95% CI: 1.56–3.89, p < 0.001). Women with annual household incomes of $200,000 or more had significantly higher odds of HPV knowledge (OR = 1.77, 95% CI: 1.16–2.69, *p* = 0.008) and HPV vaccine awareness (OR = 2.12, 95% CI: 1.18–3.79, *p* = 0.012) compared with those earning less than $50,000 annually. In addition, employed women had higher odds of HPV knowledge (OR = 1.34, 95% CI: 1.11–1.62, *p* = 0.002), while women with health insurance had higher odds of HPV vaccine awareness (OR = 1.62, 95% CI: 1.18–2.23, *p* = 0.003). Significant racial/ethnic differences were observed. Compared with non-Hispanic White women, Hispanic women had lower odds of HPV knowledge (OR = 0.62, 95% CI: 0.48–0.79, *p* < 0.001) and HPV vaccine awareness (OR = 0.45, 95% CI: 0.32–0.61, *p* < 0.001). Similarly, non-Hispanic Black women had lower odds of HPV knowledge (OR = 0.75, 95% CI: 0.59–0.95, *p* = 0.020) and HPV vaccine awareness (OR = 0.45, 95% CI: 0.33–0.61, *p* < 0.001), while non-Hispanic Asian women had substantially lower odds of HPV knowledge (OR = 0.33, 95% CI: 0.21–0.50, *p* < 0.001) and HPV vaccine awareness (OR = 0.13, 95% CI: 0.08–0.21, *p* < 0.001).

**Table 2:** Adjusted ORs^a^ and 95% CIs for HPV knowledge and HPV vaccine awareness by health-related social media engagement.

Variable	HPV knowledge, OR (95% CI)	*p-*value	HPV vaccine awareness, OR (95% CI)	*p-*value
Health-related social media engagement				
No	Ref^b^			
Yes	1.75 (1.43–2.13)	< 0.001	1.60 (1.25–2.05)	< 0.001
Age				
30–64	Ref			
21–29	0.86 (0.65–1.14)	0.302	0.79 (0.55–1.15)	0.22
Race/ethnicity				
Non-Hispanic White	Ref			
Hispanic	0.62 (0.48–0.79)	< 0.001	0.45 (0.32–0.61)	< 0.001
Non-Hispanic Black	0.75 (0.59–0.95)	0.020	0.45 (0.33–0.61)	< 0.001
Non-Hispanic Asian	0.33 (0.21–0.50)	< 0.001	0.13 (0.08–0.21)	< 0.001
Non-Hispanic Other	0.54 (0.35–0.83)	0.005	0.44 (0.25–0.78)	0.004
Education				
Less than high school	Ref			
High school/some college	1.29 (0.88–1.87)	0.189	1.38 (0.91–2.10)	0.131
College and above	3.36 (2.25–5.01)	< 0.001	2.46 (1.56–3.89)	< 0.001
Employment status				
No	Ref			
Yes	1.34 (1.11–1.62)	0.002	1.25 (0.98–1.58)	0.066
Household income				
$0–$49,999	Ref			
$50,000–$ 99,999	1.05 (0.84–1.31)	0.654	1.35 (1.02–1.79)	0.036
$100000–$199,999	1.33 (0.99–1.78)	0.050	2.00 (1.43–2.98)	0.001
$200,000+	1.77 (1.16–2.69)	0.008	2.12 (1.18–3.79)	0.012
Insurance coverage				
No	Ref			
Yes	1.28 (0.97–1.68)	0.084	1.62 (1.18–2.23)	0.003
Marital status				
No	Ref			
Yes	0.88 (0.73–1.08)	0.226	0.85 (0.66–1.10)	0.216
Region				
Northeast	Ref			
Midwest	0.95 (0.86–1.29)	0.040	0.96 (0.63–1.47)	0.732
South	0.75 (0.57–0.99)	0.260	0.82 (0.58–1.18)	0.040
West	0.84 (0.62–1.13)	0.732	0.83 (0.56–1.22)	0.260
Health status				
Poor/fair	Ref			
Good	1.09 (0.85–1.39)	0.503	1.57 (1.17–2.11)	0.003
Very good/Excellent	1.17 (0.91–1.50)	0.230	1.52 (1.12–2.07)	0.007

a Adjusted ORs: adjusted for age, education, race/ethnicity, insurance coverage, household income, marital status, health status, region, and employment status.

b Ref: Reference category.

An ordered logistic regression model revealed a significant association between the extent of health-related social media engagement and levels of HPV knowledge among women in the United States (Table 3). Compared to women with no engagement, those with increasing levels of social media engagement had progressively higher odds of greater HPV knowledge: level 1 (OR = 1.65, 95% CI: 1.31–2.07, *p* < 0.001), level 2 (OR = 1.71, 95% CI: 1.31–2.24, *p* < 0.001), level 3 (OR = 2.05, 95% CI: 1.51–2.78, *p* < 0.001), and level 4 (OR = 1.76, 95% CI: 1.28–2.43, *p* = 0.001). The largest odds ratio was observed among women with level 3 engagement, followed by those with level 4 engagement.

**Table 3:** Adjusted ORs^a^ and 95% CIs for HPV knowledge and HPV vaccine awareness by the extent of health-related social media engagement.

Variable	HPV knowledge, OR (95% CI)	*p-*value	HPV vaccine awareness, OR (95% CI)	*p-*value
Extent of health-related social media engagement				
0	Ref^b^			
1	1.65 (1.31–2.07)	< 0.001	1.48 (1.11–1.98)	0.008
2	1.71 (1.31–2.24)	< 0.001	1.81 (1.27–2.57)	0.001
3	2.05 (1.51–2.78)	< 0.001	1.57 (1.07–2.29)	0.020
4	1.76 (1.28–2.43)	0.001	1.82 (1.18–2.78)	0.006
Age				
30–64	Ref			
21–29	0.87 (0.65–1.15)	0.338	0.82 (0.56–1.19)	0.293
Race/ethnicity				
Non-Hispanic White	Ref			
Hispanic	0.62 (0.48–0.97)	< 0.001	0.44 (0.32–0.60)	< 0.001
Non-Hispanic Black	0.75 (0.59–0.96)	0.021	0.44 (0.33–0.60)	< 0.001
Non-Hispanic Asian	0.33 (0.22–0.50)	< 0.001	0.13 (0.08–0.20)	< 0.001
Non-Hispanic Other	0.54 (0.35–0.83)	0.005	0.44 (0.25–0.76)	0.003
Education				
Less than high school	Ref			
High school/some college	1.28 (0.88–1.86)	0.200	1.32 (0.86–2.01)	0.200
College and above	3.32 (2.22–4.97)	< 0.001	2.38 (1.50–3.77)	< 0.001
Employment status				
No	Ref			
Yes	1.33 (1.11–1.62)	0.002	1.21 (0.96–1.54)	0.109
Household income				
$0–$49,999	Ref			
$50,000–$ 99,999	1.05 (0.84–1.32)	0.640	1.36 (1.02–1.80)	0.035
$100000–$199,999	1.33 (0.99–1.78)	0.051	2.00 (1.34–2.99)	0.001
$200,000+	1.77 (1.16–2.69)	0.008	2.07(1.15–3.73)	0.015
Insurance coverage				
No	Ref			
Yes	1.27 (0.96–1.67)	0.093	1.64 (1.19–2.27)	0.002
Marital status				
No	Ref			
Yes	0.89 (0.73–1.08)	0.251	0.84 (0.65–1.08)	0.182
Region				
Northeast	Ref			
Midwest	0.94 (0.68–1.29)	0.708	0.97 (0.64–1.50)	0.923
South	0.75 (0.57–0.98)	0.038	0.83 (0.58–1.20)	0.327
West	0.84 (0.62–1.13)	0.253	0.84 (0.57–1.24)	0.378
Health status				
Poor/fair	Ref			
Good	1.09 (0.86–1.40)	0.465	1.57 (1.17–2.11)	0.003
Very good/Excellent	1.18 (0.91–1.51)	0.206	1.53 (1.13–2.08)	0.006

a Adjusted ORs: Adjusted for age, education, race/ethnicity, insurance coverage, household income, marital status, health status, region, and employment status.

b Ref: Reference category.

**Figure 1:**
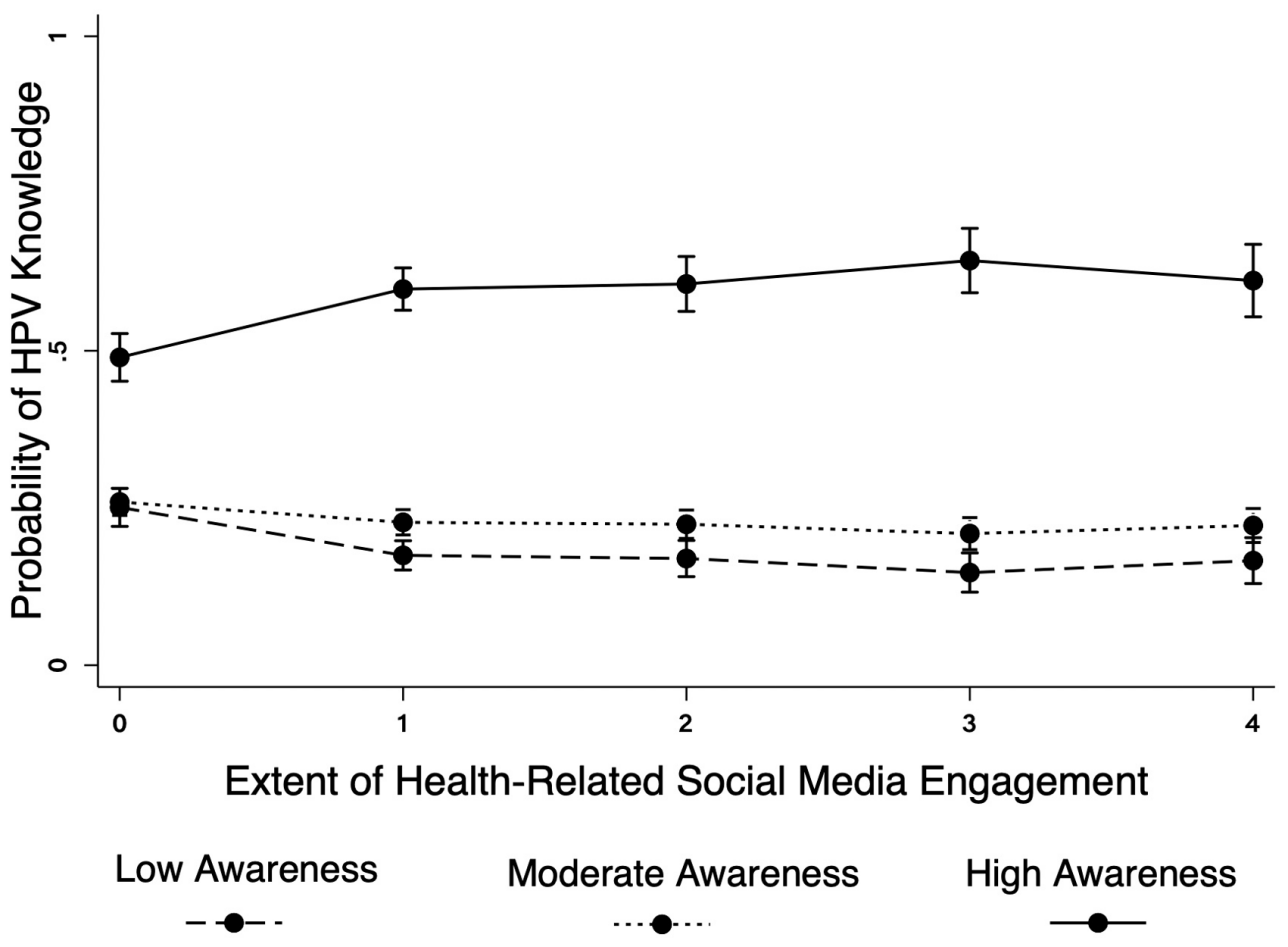
Predicted probabilities of low, moderate, and high HPV knowledge by extent of health-related social media engagement among US women aged 21–65 years. Estimates are derived from adjusted ordinal logistic regression models. Low, moderate, and high HPV knowledge correspond to increasing scores on the HPV knowledge scale.

As shown in Figure 1, increased participation in health-related social media activities was associated with higher levels of HPV knowledge, with a significant linear trend observed across increasing levels of engagement (χ^2^ = 13.87, *p* = 0.0002; Supplementary Table S4). The probability of high awareness increased steadily with greater engagement, while low awareness decreased, and moderate awareness remained relatively stable. Across all engagement levels, the probability of high HPV knowledge was consistently grater among women reporting higher social media engagement.

**Figure 2:**
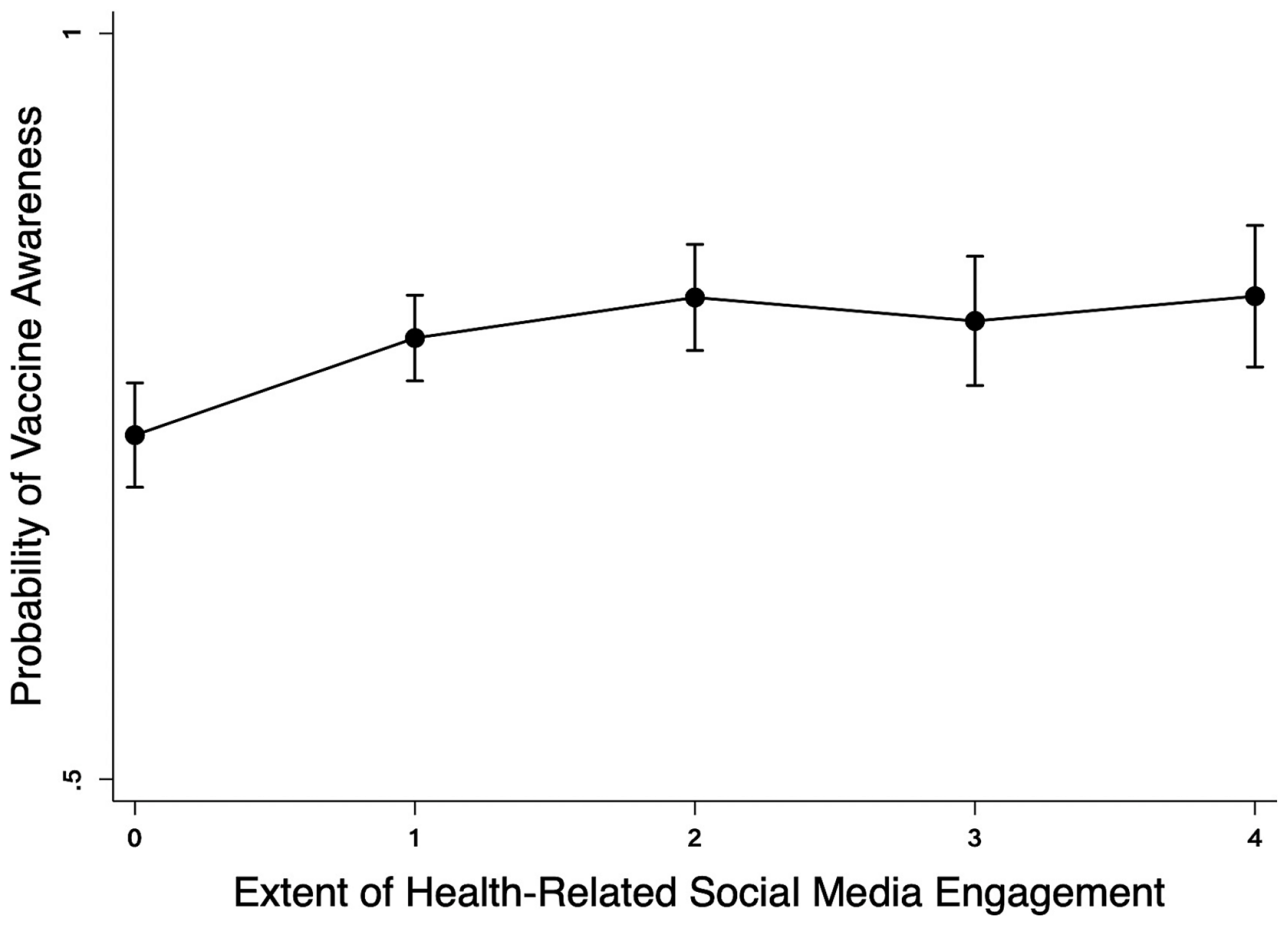
Predicted probability of HPV vaccine awareness by extent of health-related social media engagement among US women aged 21–65 years. Estimates are derived from adjusted logistic regression models. HPV vaccine awareness corresponds to awareness of the HPV vaccine (yes vs. no).

A similar pattern was observed for HPV vaccine awareness (Figure 2). Increased health-related social media engagement was associated with higher awareness, with a significant linear trend (χ^2^ = 7.85, *p* = 0.005; Supplementary Table S4). Awareness increased with initial engagement and plateaued at higher levels. The greatest increase in predicted awareness occurred between women reporting no engagement and those reporting some level of engagement.

### 3.3 Associations between cervical cancer screening (pap smear) and health-related social media engagement

Significant relationship between Health-Related Social Media Engagement and Pap Smear Utilization was found (Table 4). Based on the adjusted logistic regression model, women who engaged with health-related content on social media had significantly higher odds of Pap smear utilization compared to those who did not (OR = 1.26, 95% CI: 1.00–1.59, *p* = 0.044). Unadjusted regression model also showed a similar association (*p* ≤ 0.002; Supplementary Table S2).

**Table 4:** Adjusted ORs^a^ and 95% CIs for pap smear utilization by health-related social media engagement.

Variable	Pap smear utilization, OR (95% CI)	*p-*value
Health-related social media engagement		
No	Ref^b^	
Yes	1.26 (1.00–1.59)	0.044
Age		
30–64	Ref	
21–29	1.07 (0.77–1.47)	0.684
Race/ethnicity		
Non-Hispanic White	Ref	
Hispanic	1.29 (0.97–1.73)	0.081
Non-Hispanic Black	1.90 (1.42–2.55)	< 0.001
Non-Hispanic Asian	1.11 (0.68–1.81)	0.676
Non-Hispanic Other	1.12 (0.67–1.88)	0.655
Education		
Less than high school	Ref	
High school/some college	1.11 (0.71–1.72)	0.647
College and above	1.34 (0.84–2.15)	0.212
Employment status		
No	Ref	0.174
Yes	1.16 (0.93–1.45)	
Household income		
$0–$49,999	Ref	
$50,000–$ 99,999	1.15 (0.89–1.50)	0.277
$100000–$199,999	1.30 (0.93–1.82)	0.117
$200,000+	2.28 (1.37–3.79)	0.002
Insurance coverage		
No	Ref	
Yes	1.50 (1.10–2.05)	0.010
Marital status		
No	Ref	
Yes	1.21 (0.96–1.53)	0.093
Region		
Northeast	Ref	
Midwest	0.95 (0.66–1.36)	0.772
South	0.91 (0.67–1.25)	0.656
West	0.78 (0.55–1.10)	0.160
Health status		
Poor/fair	Ref	
Good	1.15 (0.87–1.53)	0.316
Very good/Excellent	1.44 (1.08–1.93)	0.014

a Adjusted ORs: adjusted for age, education, race/ethnicity, insurance coverage, household income, marital status, health status, region, and employment status.

b Ref: Reference category.

An adjusted logistic regression model examined the association between the extent of health-related social media engagement and Pap smear utilization among women in the United States (Table 5). Although women with any level of social media engagement had higher odds of Pap smear utilization compared to those with no engagement, the association reached statistical significance only at the higher engagement levels. Specifically, women with level 3 and level 4 engagement had significantly greater odds of Pap smear utilization (level 3: OR = 1.42, 95% CI: 1.00–2.00, *p* = 0.048; level 4: OR = 1.77, 95% CI: 1.19–2.63, *p* = 0.005). The unadjusted logistic regression models showed similar results (Supplementary Table S3). Among all engagement categories, women with level 4 engagement demonstrated the highest odds of Pap smear utilization.

**Table 5:** Adjusted ORs^a^ and 95% CIs for pap smear by the extent of health-related social media engagement.

Variable	Pap smear utilization, OR (95% CI)	*p-*value
Extent of health-related social media engagement		
0	Ref^b^	
1	1.12 (0.86–1.47)	0.395
2	1.23(0.90–1.67)	0.198
3	1.42 (1.00–2.00)	0.048
4	1.77 (1.19–2.63)	0.005
Age		
30–64	Ref	
21–29	1.10 (0.79–1.51)	0.572
Race/ethnicity		
Non-Hispanic White	Ref	
Hispanic	1.32 (0.98–1.76)	0.064
Non-Hispanic Black	1.94 (1.44–2.60)	< 0.001
Non-Hispanic Asian	1.14 (0.70–1.86)	0.604
Non-Hispanic Other	1.15 (0.68–1.92)	0.603
Education		
Less than high school	Ref	
High school/some college	1.07 (0.68–1.67)	0.763
College and above	1.29 (0.80–2.08)	0.282
Employment status		
No	Ref	
Yes	1.16 (0.92–1.44)	0.201
Household income		
$0–$49,999	Ref	
$50,000–$ 99,999	1.16 (0.89–1.50)	0.263
$100000–$199,999	1.30 (0.94–1.84)	0.110
$200,000+	2.18 (1.30–3.64)	0.003
Insurance coverage		
No	Ref	
Yes	1.50 (1.09–2.04)	0.011
Marital status		
No	Ref	
Yes	1.22 (0.96–1.53)	0.090
Region		
Northeast	Ref	
Midwest	0.95 (0.66–1.36)	0.776
South	0.91 (0.66–1.25)	0.567
West	0.78 (0.55–1.10)	0.157
Health status		
Poor/fair	Ref	
Good	1.17 (0.88–1.55)	0.270
Very good/Excellent	1.22 (1.10–1.98)	0.009

a Adjusted ORs: Adjusted for age, education, race/ethnicity, insurance coverage, household income, marital status, health status, region, and employment status.

b Ref: Reference category.

**Figure 3:**
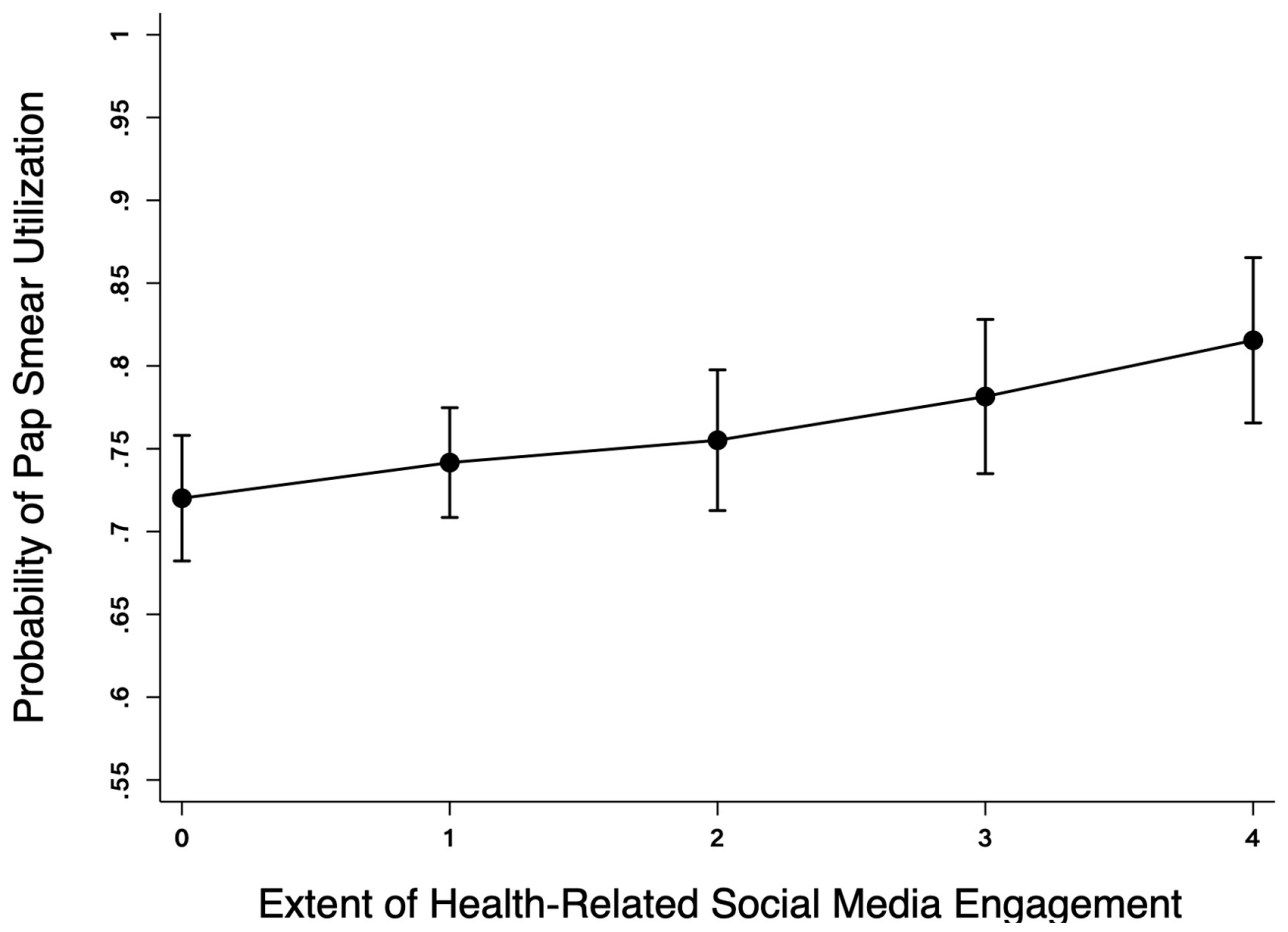
Predicted probability of Pap smear utilization by extent of health-related social media engagement among US women aged 21–65 years. Estimates are derived from adjusted logistic regression models. Pap smear utilization corresponds to self-reported history of Pap smear screening (yes vs. no).

Figure 3 shows that greater health-related social media engagement was also associated with higher Pap smear utilization. A significant linear trend was observed (χ^2^ = 9.42, *p* = 0.0021; Supplementary Table S4), with predicted probabilities increasing progressively from no engagement to higher engagement levels, indicating a dose–response relationship with cervical cancer screening. The highest predicted probability of Pap smear utilization was observed among women reporting level 4 engagement.

### 3.4 Effect modification by race/ethnicity

A significant interaction was observed between health-related social media engagement and race/ethnicity in relation to Pap smear utilization (Table 6). While engagement in health-related social media was generally associated with higher odds of Pap smear utilization (OR = 1.46, 95% CI:1.08–1.99, *p* = 0.015), this effect varied significantly by ethnic group. Specifically, the interaction term for Hispanic women indicated a reduced effect of social media engagement (OR = 0.41, 95% CI: 0.22–0.79, *p* = 0.007), suggesting that the positive association observed in the overall sample was weaker among Hispanic women. No statistically significant interaction effects were found for other racial/ethnic groups. Interaction terms for non-Hispanic Black, non-Hispanic Asian, and non-Hispanic Other women were not statistically significant.

**Table 6:** Coefficients and 95% CIs for pap smear utilization by health-related social media engagement, effect modified by race/ethnicity.

Pap smear utilization	Coefficient (95% CI)	*p*
Health-related social media engagement		
No	Ref^a^	
Yes	1.46 (1.08–1.99)	0.015
Race/ethnicity		
Non-Hispanic White	Ref	
Hispanic	3.75 (2.09–6.72)	<0.001
Non-Hispanic Black	1.06 (0.63–1.76)	0.834
Non-Hispanic Asian	2.22 (0.59–8.25)	0.235
Non-Hispanic Other	1.36 (0.44–4.22)	0.590
Effect modification by race/ethnicity		
Hispanic: Engaged vs. Not engaged	0.41 (0.22–0.79)	0.007
Non-Hispanic Black: Engaged vs. Not engaged	1.32 (0.72–2.41)	0.365
Non-Hispanic Asian: Engaged vs. Not engaged	0.44 (0.11–1.80)	0.256
Non-Hispanic Other: Engaged vs. Not engaged	0.77 (0.22–2.74)	0.687

a Ref: Reference category.

## 4 Discussion

This study examined the association between health-related social media engagement and women’s HPV knowledge, HPV vaccine awareness, and cervical cancer screening utilization in a large, nationally representative sample of US women aged 21–65 years. Overall, more than two-thirds of women reported participating in at least one health-related activity on social media, with watching health-related videos being the most common form of engagement. Despite high levels of social media use, substantial sociodemographic disparities persisted, particularly across racial/ethnic groups.

Engagement with health-related content on social media was significantly associated with higher HPV knowledge and vaccine awareness, consistent with prior research showing that interacting with accurate online health information improves health literacy and preventive health behaviors. For example, Zhao and Zhang found that exposure to HPV-related information on social networking sites was strongly associated with greater HPV knowledge and vaccine intentions among young women, while Ortiz et al. reported that women who used social media for health information demonstrated higher awareness of HPV vaccination guidelines (18, 19). Similarly, Vraga and Bode showed that interacting with accurate health content on social media improves health literacy and reduces misconceptions about HPV (20). These patterns align with our findings that women who interacted with health information on social media had 75% higher odds of possessing greater HPV knowledge and 60% higher odds of vaccine awareness (*p* < 0.001 for both models). These findings suggest that social media platforms may serve as important channels for disseminating HPV-related information and increasing awareness of preventive behaviors. Women who actively seek or engage with online health content may be more health conscious, more informed about HPV-related risks, and better equipped to make informed decisions about vaccination and screening (20, 21). However, not all studies support the positive association observed in our analysis. Nan and Madden reported no significant relationship between online HPV information exposure and vaccination attitudes, suggesting that digital engagement may be insufficient when information quality is low or when trust in online sources is limited (22).

Engagement with health-related social media was also associated with higher odds of Pap smear utilization, although the magnitude of the association was modest. Women who engaged in these activities had 26% higher odds of reporting Pap smear utilization compared with non-engaged women. Moreover, statistically significant associations were observed only among women with the highest levels of engagement (levels 3 and 4), whereas lower engagement levels were not significantly associated with Pap smear utilization. This finding suggests that the relationship between health-related social media engagement and screening behavior may be less consistent than that observed for HPV knowledge and vaccine awareness. This pattern aligns with previous research showing that online health information seeking is positively associated with cervical cancer screening and that active engagement with health content on social media supports participation in preventive services (23, 24). However, Qin et al. reported no association between general, non–health-specific social media use and screening behaviors such as pap smear (25).

While health-related social media engagement was positively associated with HPV knowledge, HPV vaccine awareness, and Pap smear utilization, the quality and accuracy of information available on social media platforms vary considerably. Exposure to misinformation regarding HPV vaccination, cervical cancer risk, or screening recommendations may undermine preventive behaviors and contribute to vaccine hesitancy or delayed screening (26). Therefore, public health organizations, healthcare providers, and community stakeholders should prioritize the dissemination of evidence-based and culturally appropriate information through trusted digital channels while actively addressing misinformation. Strategies such as fact-checking initiatives, partnerships with healthcare professionals, and targeted health communication campaigns may help improve the reliability of online health information and strengthen public trust (27).

In addition, reliance on social media as a health communication strategy may not adequately reach populations with limited internet access, lower digital literacy, or reduced engagement with digital technologies (28). Individuals living in underserved communities, rural areas, or economically disadvantaged settings may face barriers to accessing online health information. Consequently, social media–based interventions should be complemented by community outreach programs, healthcare provider recommendations, public health campaigns, and other traditional communication approaches to ensure equitable access to cervical cancer prevention information across diverse populations.

Our results demonstrated a clear dose–response relationship between health-related social media engagement and HPV knowledge or vaccine awareness. As the number of engagement activities increased, the likelihood of high HPV knowledge rose steadily, peaking among those engaging in three to four distinct health-related social media behaviors. A similar pattern was observed for HPV vaccine awareness, although awareness plateaued after two engagement activities. These graded associations align with previous research showing that multidimensional engagement with digital health tools enhances health literacy and promotes preventive behaviors (29, 30). Lama et al. similarly found that, compared to non-users, greater engagement in social-media behaviors was associated with higher HPV awareness, supporting our finding that even low-level engagement positively influences knowledge (31). Overall, the positive linear trends across engagement levels were most evident for HPV knowledge and vaccine awareness, while associations with Pap smear utilization were comparatively weaker.

Despite the benefits of social media engagement, our analysis revealed marked racial and ethnic disparities across all HPV-related outcomes. Compared to non-Hispanic White women, non-Hispanic Black, Asian, Hispanic, and Other racial groups consistently exhibited significantly lower odds of HPV knowledge and vaccine awareness, even after adjusting for socioeconomic, health, and demographic characteristics (Tables 2, 3). For instance, for HPV knowledge, non-Hispanic Black (OR = 0.75, 95% CI: 0.59–0.95, *p* = 0.020), non-Hispanic Asian (OR = 0.33, 95% CI: 0.21–0.50, *p* < 0.001), Hispanic (OR = 0.62, 95% CI: 0.48–0.79, *p* < 0.001), and non-Hispanic Other (OR = 0.54, 95% CI: 0.35–0.83, *p* = 0.005) women had the lowest odds (Table 2).

Similarly, for HPV vaccine awareness, non-Hispanic Black (OR = 0.45, 95% CI: 0.33–0.61, *p* < 0.001), non-Hispanic Asian (OR = 0.13, 95% CI: 0.08–0.21, *p* < 0.001), Hispanic (OR = 0.45, 95% CI: 0.32–0.61, *p* < 0.001), and non-Hispanic Other (OR = 0.44, 95% CI: 0.25–0.78, *p* = 0.004) women showed substantially reduced odds (Table 2).

Racial and ethnic differences were also observed in Pap smear utilization (Tables 4, 5). For example, Non-Hispanic Black women had significantly higher odds of screening compared to non-Hispanic White women (OR = 1.90, 95% CI: 1.42–2.55, *p* < 0.001; Table 4). However, evidence from a meta-analysis indicates that, overall, Black women generally have lower access to Pap smear screening compared to White women, although differences between Black women and other ethnic groups (except White) were not significant (32). These findings suggest that while our sample observed higher screening among non-Hispanic Black women, broader population-level data highlight persistent disparities that may depend on context, region, and access to care.

Effect modification analyses further revealed that the positive association between social media engagement and Pap smear utilization was significantly weaker among Hispanic women (*p* = 0.007; Table 6). This suggests that while health-oriented social media engagement may promote screening behavior in the general population, its effect varies across racial and ethnic groups. Cultural preferences, variations in media consumption, and differences in healthcare access may explain why Hispanic women derive less benefit from social media engagement in this context (33). These findings highlight persistent inequities in access to or retention of HPV-related information. Factors such as differences in digital literacy, access to culturally appropriate online content, and trust in health information sources may contribute to these disparities (30–32). Collectively, these results underscore the ongoing need to tailor digital health communication strategies to effectively reach historically underserved populations.

This study has several limitations. First, the cross-sectional design of HINTS limits the ability to establish causal relationships between health-related social media engagement and HPV knowledge, HPV vaccine awareness, or Pap smear utilization. The observed associations may reflect reverse causation, whereby women who are already more health-conscious and engaged in preventive health behaviors are more likely to seek health information online. Additionally, residual confounding cannot be excluded. Second, all measures were self-reported and may be subject to recall bias or social desirability bias. Third, the proportional odds assumption underlying the ordinal logistic regression models was not formally tested. However, the ordered logistic model was considered appropriate given the ordinal nature of the outcome variable. Fourth, the study assessed engagement with health-related content on social media but did not evaluate the accuracy, quality, or source of the information encountered. Finally, disparities in internet access, digital literacy, and social media use were not directly assessed.

The study’s findings collectively highlight the positive association between health-related social media engagement and HPV-related knowledge and vaccine awareness, and to a lesser extent cervical cancer screening utilization, while also emphasizing the persistence of racial and ethnic disparities in these outcomes. Efforts to enhance the reach and effectiveness of digital health communication should prioritize culturally tailored messaging, improve digital literacy programs, and expand access to reliable online health information. Interventions that promote more frequent and active engagement with credible health content may yield meaningful improvements in women’s preventive health behaviors. However, the observed association with Pap smear utilization was modest compared with HPV knowledge and vaccine awareness. Future research is needed to further understand how specific forms of social media engagement influence women’s health behaviors and to identify strategies to reduce disparities in digital health benefits. Intervention studies such as targeted online educational campaigns or community-based digital literacy programs may be particularly valuable for reaching underserved populations.

## Data Availability

The original contributions presented in the study are included in the article/Supplementary material, further inquiries can be directed to the corresponding author.
