# Supplementary material for: Health-related social media engagement and cervical cancer prevention: associations with HPV knowledge, vaccine awareness, and pap smear utilization among US women
Source: Front Public Health. 2026 Jul 7;14:1782691. doi: 10.3389/fpubh.2026.1782691 (PMC13385090; doi:10.3389/fpubh.2026.1782691)
Supplement: Supplementary file 1 [file Table_1.DOCX]

Supplementary Material

Table S 1. Frequency and percentage of health-related engagement activities on social media among women aged 21–65 (n = 2,247)

| **Variable** | **Frequency (N), Percentage (%)** |
| --- | --- |
| Sharing personal health information  No  Yes | 1821 (81.04)  426 (18.96) |
| Sharing general health-related information  No  Yes | 1432 (63.81)  812 (36.19) |
| Interacting with Peers who have similar health or medical issues  No  Yes | 1601 (71.25)  646 (28.75) |
| Watching health-related video  No  Yes | 762 (33.91)  1485 (66.09) |

Table S2. Unadjusted ORs and 95% CIs for HPV Knowledge, HPV Vaccine Awareness, and Pap smear utilization by Health-Related Social Media Engagement

| **Variable** | **HPV Knowledge**  ***OR (95% CI) p*** | **HPV Vaccine Awareness**  ***OR (95% CI), p*** | **Pap Smear Utilization**  ***OR (95% CI), p*** |
| --- | --- | --- | --- |
| Health-Related SM Engagement  No  Yes | Reference  2.05 (1.72 – 2.46),*p* <0.001 | Reference  1.85 (1.49 – 2.29), *p* <0.001 | Reference  1.40 (1.14 – 1.74), *p*=002 |

Table S3. Unadjusted ORs and 95% CIs for HPV Knowledge, HPV Awareness, and Pap Smear by the Extent of Health-Related Social Media Engagement

| **Variable** | **HPV Knowledge**  ***OR (95% CI) p*** | **HPV Vaccine Awareness**  ***OR (95% CI), p*** | **Pap Smear Utilization**  ***OR (95% CI), p*** |
| --- | --- | --- | --- |
| Extent of Health-Related SM Engagement  0  1  2  3  4 | Reference  1.89 (1.53 – 2.33), *p* < 0.001  2.08 (1.62 – 2.65), *p* < 0.001  2.64 (2.00 – 3.49), *p* < 0.001  1.90 (1.41 – 2.57), *p* < 0.001 | Reference  1.67 (1.29 – 2.15), *p* <0.001  2.03 (1.50 – 2.76), *p* <0.001  2.10 (1.49 – 2.96), *p* <0.001  1.83 (1.26 – 2.67), *p=* 0.002 | Reference  1.31 (1.02 – 1.68), *p=* 0.034  1.33 (0.99 – 1.77), *p*= 0.054  1.59 (1.15 – 2.21), *p=* 0.005  1.67 (1.15 – 2.42), *p=* 0.007 |

Table S4. Linear Trend Tests for the Association Between Extent of Health-Related Social Media Engagement and HPV Awareness, HPV Vaccine Awareness, and Pap Smear Utilization

| Extent of Health-Related SM Engagement  (Linear) | Degree of Freedom | Chi2 | ***p*** |
| --- | --- | --- | --- |
|  | **HPV Awareness** | | |
|  | 1 | 13.87 | 0.0002 |
|  | **Vaccine Awareness** | | |
|  | 1 | 7.85 | 0.0050 |
|  | **Pap Smear Utilization** | | |
|  | 1 | 9.42 | 0.0021 |
